# DNA barcode analysis: a comparison of phylogenetic and statistical classification methods

**DOI:** 10.1186/1471-2105-10-S14-S10

**Published:** 2009-11-10

**Authors:** Frederic Austerlitz, Olivier David, Brigitte Schaeffer, Kevin Bleakley, Madalina Olteanu, Raphael Leblois, Michel Veuille, Catherine Laredo

**Affiliations:** 1 CNRS, Laboratoire Ecologie Systématique et Evolution, UMR 8079, Orsay, F-91405; Univ Paris-Sud, Orsay, F-91405; AgroParisTech, Paris, F-75231, France; 2 UR341, Mathématiques et informatique appliquées, INRA, F-78350 Jouy-en-Josas, France; 3 Muséum National d'Histoire Naturelle, UMR 5202 MNHN/CNRS, Laboratoire Origine Structure Evolution de la Biodiversité, 16 rue Buffon, 75005 Paris, France; 4 Laboratoire de Biologie intégrative des populations, Ecole Pratique des Hautes Etudes, Paris, France; 5 Institut Curie, Centre de Recherche, Paris, F-75248 France; 6 INSERM, U900, Paris, F-75248 France; 7 Centre for Computational Biology, Ecole des Mines de Paris, 35 rue St Honoré, Fontainebleau, F-77305 France; 8 Laboratoire de Probabilités et Modèles Aléatoires, Universités Paris 6 et 7, UMR CNRS 7599, 4 place Jussieu, 75005 Paris, France

## Abstract

**Background:**

DNA barcoding aims to assign individuals to given species according to their sequence at a small locus, generally part of the CO1 mitochondrial gene. Amongst other issues, this raises the question of how to deal with within-species genetic variability and potential transpecific polymorphism. In this context, we examine several assignation methods belonging to two main categories: (i) phylogenetic methods (neighbour-joining and PhyML) that attempt to account for the genealogical framework of DNA evolution and (ii) supervised classification methods (k-nearest neighbour, CART, random forest and kernel methods). These methods range from basic to elaborate. We investigated the ability of each method to correctly classify query sequences drawn from samples of related species using both simulated and real data. Simulated data sets were generated using coalescent simulations in which we varied the genealogical history, mutation parameter, sample size and number of species.

**Results:**

No method was found to be the best in all cases. The simplest method of all, "one nearest neighbour", was found to be the most reliable with respect to changes in the parameters of the data sets. The parameter most influencing the performance of the various methods was molecular diversity of the data. Addition of genetically independent loci - nuclear genes - improved the predictive performance of most methods.

**Conclusion:**

The study implies that taxonomists can influence the quality of their analyses either by choosing a method best-adapted to the configuration of their sample, or, given a certain method, increasing the sample size or altering the amount of molecular diversity. This can be achieved either by sequencing more mtDNA or by sequencing additional nuclear genes. In the latter case, they may also have to modify their data analysis method.

## Introduction

Hebert *et al. *[1] defined the DNA barcode as a short sequence used as a standard tool to identify the species to which an organism belongs. Its purpose is to provide a simple and automatic method to correctly identify species, with little or no recourse to taxonomic expertise. The 5' half of the cytochrome *c *oxydase I (COI) mtDNA gene has been chosen as the barcode locus for most animals, and gene markers with similar barcoding properties have been investigated in plants, fungi and protists. The approach has been successfully applied to various kinds of organisms [2-4], although problems have arisen in some cases. For instance, barcoding can be less successful in the case of paraphyly [5,6]. Moreover, horizontal transfer of mitochondria together with Wolbachia across species [7] can make the COI locus ineffective.

Recently separated species will be the more difficult to distinguish with barcoding techniques. Indeed, these species may share many polymorphic sites that were polymorphic in the ancestral species. Time will be needed for these polymorphic sites to be fixed and for specific mutations to appear in each species. The number of mutations separating two individuals for the COI locus increases with their coalescence time, but since this locus is not itself the cause of speciation events, no clear-cut change is expected to occur in its variation when crossing the border leading from one species to another. Hence, one important issue is to interpret data using methods that will minimise the probability of an incorrect conclusion.

In the simplest application of DNA barcoding, a reference data set from a given group of organisms (a genus or family) is constructed from the DNA barcode sequences of a reference sample of individuals known to belong to already described species. Then, query sequences of individuals from this group, but of unknown taxonomic status, are matched to this reference data set and data analysis consists in assigning these individuals to one of the given species [see e.g., the Barcode Of Life Data system, BOLD, [8]]. Furthermore, specific analyses are needed to detect potentially new species using their barcode. We focus here on cases in which the query individual is already characterized at a given taxonomic level (e.g., family), for which a reference sample is available. Usually this attribution at high taxonomic level can be done either directly through phenotyping methods or through an initial BLAST procedure on the databases.

**Figure 1:**
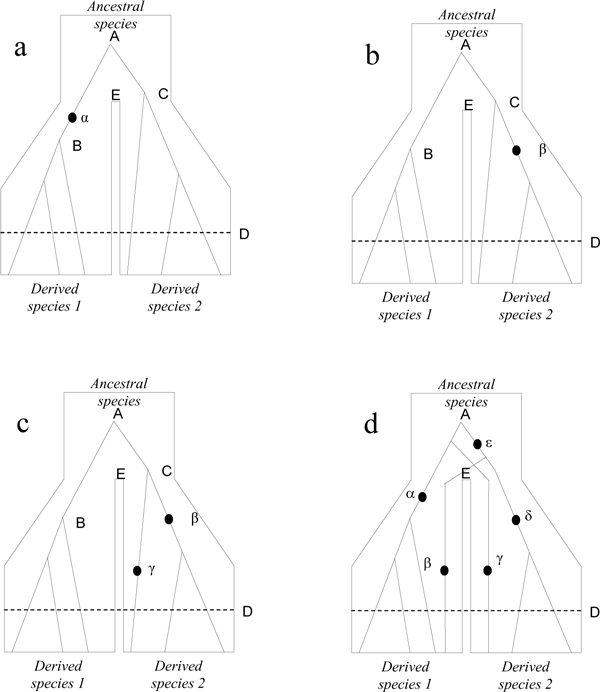
**Hypothetical representations of gene genealogies between two species and of some hypothetical mutation patterns between them**. Individual A is the global MRCA of all individuals; individuals B and C are respectively the MRCA of the two derived species 1 and 2. Cases a, b and c correspond to reciprocal monophyly and case d to reciprocal paraphyly. In some cases of reciprocal monophyly, one mutation is diagnostic (a), while no mutation is diagnostic in other cases (b and c). A combination of mutation can also be sufficient to perform barcoding (c). Barcoding is also possible in the case of reciprocal paraphyly, by also using combinations of mutations that are specific to a given species (d).

Figure 1 shows several cases that may occur in the barcoding context. In the simplest case (Figure 1a), a species differs from all others by a diagnostic mutation (*α*), i.e., a mutation present only in all individuals of one species, but in none of the other species. This is only possible in cases of reciprocal monophyly, where the most common recent ancestor (MRCA) of all individuals in each species (individuals B and C in Figure 1a-c) is more recent than the global MRCA (individual A). Second, to be diagnostic for a given species, a mutation must occur in the lineage leading to the MRCA of this species, as for example mutation *α *in Figure 1a.

Thus, a new mutation may or may not, by chance, be diagnostic. For instance, mutation *α *is diagnostic in Figure 1a, whereas mutation *β *in Figure 1b is not, as it occurred after the MRCA of species 2. Also, an apparently diagnostic mutation may not remain so if a larger sample is collected. In Figure 1c, a smaller sample may have suggested mutation *β *to be diagnostic, but it is not when considering the whole sample. A simple rule of molecular population genetics states that in a sample of *n *sequences, the probability that the genealogy of the sample includes the last common ancestor of the whole species is *p *≈ (*n*-1)/(*n*+1). Thus, our confidence in having obtained a representative sample of the species variation increases with sample size, but will never reach the value of one.

It should be noted that barcoding is possible even without diagnostic mutations. In Fig. 1c, mutations *γ *and *β *together contribute to characterize species 2, though neither of them is fixed. Even in absence of reciprocal monophyly (incomplete lineage sorting, Fig. 1d), barcoding remains possible. Here, mutations α and *β *characterize species 1 whereas mutations *γ *and *δ *characterize species 2. All four mutations can thus be used in the barcoding procedure, but not mutation *ε *which is present in some individuals of both species. These last two cases explain why different data analysis methods may perform differently on different data sets, as they are based on different rationale to extract the best information from the reference sample.

The routine data analysis procedure used by BOLD [8] combines similarity methods with distance tree construction. First, the query sequence is aligned to the global alignment through a Hidden Markov Model (HMM) profile of the COI protein [9], followed by a linear search of the reference library. The 100 best hits are selected as a pre-set of "closely related tagged-specimens". Second, a Neighbour-Joining tree using the Kimura 2-parameter (K2P) distance is reconstructed on both this set and the query sequence in order to assess the relationship between the query sequence and its neighbouring reference sequences [10].

Barcoding methods can be divided into four categories: (i) similarity methods based on the match between the query sequence and the reference sequences (e.g., BLAST search); (ii) classical phylogenetic approaches like neighbour-joining [11] or maximum likelihood/Bayesian algorithms [1][5]; (iii) k-nearest neighbour based on the K2P distance and statistical approaches based on classification algorithms with no underlying biological models [e.g., CAOS, [12]]; and (iv) genealogical methods [13-15] based on coalescent theory using maximum likelihood/Bayesian algorithms based on Monte Carlo Markov Chains (MCMC). An important question is whether a realistic biological, populational or phylogenetic model for DNA barcode sequence analysis is necessary. For example, purely statistical approaches may also be able to efficiently assign query sequences to species names.

The performance of several methods belonging to the first two categories were compared in another simulation study [16]. This study focused on barcoding based on a single mitochondrial locus as in the standard procedure [8]. It showed that there was generally no best-performing method, i.e., a given method could perform better than another for a given evolutionary scenario and the reverse could be true in another. In the present paper, we compare these methods with supervised statistical classification methods. In order to consider a wide range of methods, we retained six in total: two phylogenetic methods (neighbour-joining NJ [11] and PhyML [17]), one distance method (k-nearest neighbour, k-NN), and three supervised statistical classification methods: classification and regression trees (CART), random forest (RF) and kernel methods. We ran them on two kinds of data: simulated and real [4-6]. We did not consider coalescent-based MCMC methods as they were too computer intensive to be used in a simulation process requiring many replications. Moreover they require finely-tuned parameters (e.g., length of MCMC chains) to work properly and therefore could not be run comparatively on a large number of simulated samples.

We chose to put effort into varying conditions. This included the amount of sequence information (*θ *ranging from 3 to 30), time since the split between the species (from *T *= 100 to 10,000), number of species (from 2 to 5) and sample size per species (from 3 to 25). For practical reasons, barcode reference samples generally include between 5 and 10 individuals. As noted above, a sample of 10 individuals has a probability *p *≈ (*n*-1)/(*n*+1) = 0.87 of including the MRCA of a genealogy. Thus, we could not exclude that the gain in information brought about by a larger sample would influence differently analysis methods based on very different rationales. We therefore considered different sample sizes, from 3 to 25 individuals per species. Finally, since efficiency of DNA barcoding based on the mitochondrial COI gene alone has been questioned in some cases [5,7], we also tested the performance of the various methods when including nuclear loci as additional information.

## Materials and methods

### Simulated data sets

**Figure 2:**
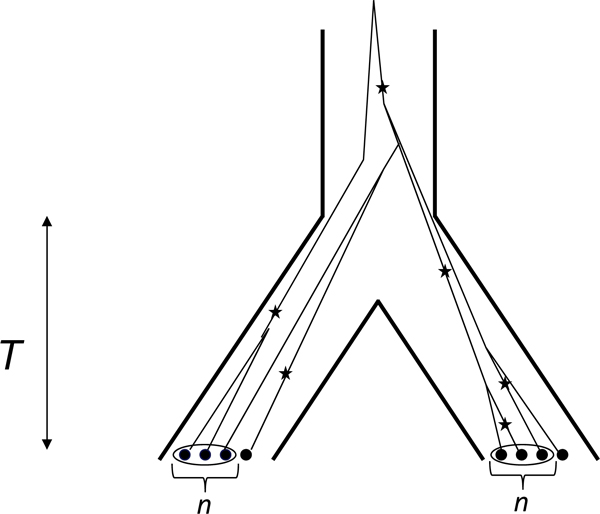
**Schematic representation of simulations for two species (*n*_S _= 2)**. It is assumed that the species split *T *generations ago. The thin lines represent the coalescent lineages and stars indicate the mutations that occurred along these lineages. For each species, we simulated *n *reference individuals and one additional individual, which was used to test the methods.

In all simulations, we considered *n*_s _species consisting each of a single panmictic population. Each population was assumed to consist of *N*_f _females and *N*_m _males, with *N*_f _= *N*_m _= 1000, amounting to a total population size *N *= *N*_f_+*N*_m _= 2000. These species were assumed to have split simultaneously *T *generations ago from a single ancestral species of size *N *(see Figure 2 for a schematic representation of the case *n*_S _= 2). An "individual" is represented by its sequence at the "barcode" locus (a mitochondrial locus, with mutation rate *μ*_c_) and a given number *n*_l _of nuclear loci (with mutation rate *μ*_n_), assuming full independence among loci. Individuals were assumed to be diploids with separated sexes, a sex ratio of 1:1 and a strictly maternal transmission of mitochondria. Hence, the population size was *N *diploid individuals for the nuclear loci (i.e., 2*N *chromosomes) and *N*_f _= *N/2 *haploid individuals (i.e., *N*/2 chromosomes) for the cytoplasmic locus. The population mutation parameter at each locus was thus *θ*_*c *_= *Nμ*_*c *_for mitochondria and *θ*_*n *_= 4*Nμ*_*n *_for nuclear loci. In all cases, we assumed *μ*_*c *_= 4*μ*_*n *_so that *θ *_*c *_= *θ*_*n*_, which we will simply denote *θ *hereafter.

For each set of parameter values, we simulated a sample of *n*+1 individual sequences in each species using the software Simcoal 2.1.2 [18]. As a first step, this software simulates backwards in time the neutral coalescent process of the different lineages relating sampled sequences to their common ancestor. In the second step, it simulates mutations occurring on the different lineages. From the *n*+1 sequences of each species, *n *sequences (hereafter referred to as the "reference sample") were used as the training data set (i.e., sequences for which the species was known) and the last sequence (*n*+1^th^) was used as the "query" (the sequence of an individual of unknown taxonomical status). However, for the change in the effective population size number, coalescent simulations were carried out the same way in the mitochondrial locus (the "barcode") and in nuclear loci, meaning that no intragenic recombination was assumed in the latter. This refinement would not have added much to the study, and the choice of some value for the non-zero recombination rate would have been arbitrary.

Five variable parameters were used. The mutation parameter had two possible values: *θ *= 3 and *θ *= 30; the reference sample size (*n*) had four possible values: 3, 5, 10 and 25; the time *T *since the split of the species took five values ranging from 100 generations (*N*_f_/10) to 10000 generations (10*N*_f_); the number of species (*n*_s_) ranged from 2 to 5; the number of nuclear loci (*n*_l_) from 0 to 4. For the mutation process, we used a Kimura two-parameter (K2P) mutation model [19] with a transition/transversion ratio of 9:1 and a uniform mutation rate over sites. A thousand simulations were run for each condition.

### Real-life data sets

We used three published data sets (Table 1) on the genus *Astraptes *[4], the cowries family [6] and a subfamily of Amazonian butterflies [5]. For cowries, we considered both the species and subspecies levels, when subspecies was known. The available locus data for *Astraptes *and cowries was the barcode (COI gene). In Amazonian butterflies, data included three loci: the COI barcode, a larger mitochondrial fragment (CoI + CoII + Leucine-tRNA) and a nuclear gene (Ef1α). We could not however combine the mitochondrial and nuclear data since the Ef1α sequences were available only for a subset of the individuals. The three data sets differed in size, in the number of species to classify and in the mutation parameter estimate *θ *(see Table 1). Experimental data sets differed from simulated data sets as they included missing data and involved many species, ranging from 12 to 180, thus leading to situations which were not all met in simulations.

**Table 1:** Characteristics of the data sets used in this study.

**Data set**	**DNA marker**	***N *sample**	***n *taxa **^*a*^	***θ***^*b*^	**Species determination**
*Astraptes*	COI (barcode)	466	12	15.32	Phylogenetical

Cowries species	COI (barcode)	2036	180	36.36	Genetical and morphological
	
Cowries species and subspecies	COI (barcode)	2036	249	36.36	

Amazonian Butterflies	COI (barcode)	424	61	46.13	Morphological
	
Amazonian Butterflies	mt DNA (CoI+ CoII Leu-t RNA)	424	61	146.09	
	
Amazonian Butterflies	nuclear (Ef1α)	191	52	57.32	

^*a *^Number of species or subspecies with at least two individuals.

^*b *^Average *θ *value per species estimated with Watterson's [37] estimate.

All six assignment methods were run on these real data sets. A leave-one-out procedure was applied in each case, which consisted in removing each individual in turn from the reference sample. The assignment method was then applied to this individual using the rest of the reference sample as a training sample. The performance of methods was evaluated as the rate at which the query individuals were successfully assigned to their species or subspecies. Individuals from species or subspecies represented by a single specimen were not used as queries in this procedure, but were retained in the training data set. For all these methods, a majority rule was used for assigning a species to query sequences. In statistical learning, it corresponded to choose a Bayes classifier associated with the 0-1 loss function [20].

### Neighbour-joining (NJ)

**Figure 3:**
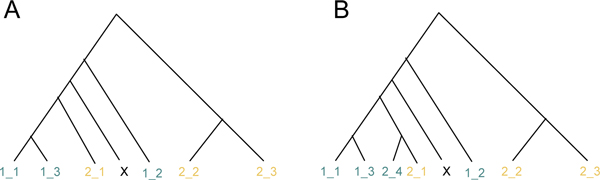
**Illustration of our assignment technique for the phylogeny-based methods**. X denotes the query sequence to assign, and individuals 1_x or 2_x belong respectively to species 1 or 2. In case A, the sister group (1_1, 1_3, 2_1) of X contains a majority of individuals of species 1, thus X is assigned to species 1. In case B, the sister group (1_1, 1_3, 2_1, 2_4) of X contains an equal number of individuals of species 1 and 2, thus we have to consider the sister group at the upper level (one node above), this group is (1_1, 1_3, 2_1, 2_4, 1_2) and contains a majority of species 1 individuals. X is thus assigned to species 1.

Neighbour-joining is a phylogenetic method consisting in constructing a tree from a distance matrix [11]. For each data set, the trees were built using the reference sample and the queries together, with the implementation provided by the APE package [21] in R [22], assuming a K2P distance between sequences. After completion of the tree, the query was assigned to the most numerous species among the members of its sister-group (Figure 3). When the two most represented species were in equal number in this group, the sister group at the upper level was used, and the same majority rule was again applied, and so on whilst equality remained. If no majority finally emerged, the result was classified as ambiguous. This very occasionally occurred in the simulated data sets and never occurred in the real data sets.

### PhyML

This alternative phylogenetic method [17] is based on maximum likelihood. We used the function *phyml_test *provided in APE that allows selection of the mutation model with the lowest Akaike information criterion (AIC) value. The tree was used as a decision criterion using the same majority rule as described for neighbour-joining.

### k-nearest neighbour classification (k-NN)

We developed an R function to apply this method [23] to DNA sequences. The method was used to rank the sequences of the training set by their closeness to the query sequence using the K2P distance computed using the *dist.dna *function of APE. Following this, the query was classified as belonging to the species including the largest number of its *k *closest neighbours, where *k *= 1, 2, 3... represents a value chosen by the user. If two or more sequences were located at the same distance from the query, both of them were included in the set of neighbours. When two species were in equal number among the *k*-nearest neighbours, the sequence was classified at random as belonging to one of these species. Three values of *k *(*k *= 1, 2, 3) were initially considered. However, as the method always performed best with *k *= 1 (1-NN), results are provided here only for this case.

### Classification and regression trees (CART)

This method [24] constructs a binary tree based on a learning sample (here the reference sequences). At the root level, all DNA sequences are put in the same class. At each step, a node is partitioned into two sub-nodes following a splitting rule based on the allelic state of the reference sequences at a given site. For each node *t*, if *p*_*j *_denotes the proportion of sequences belonging to species *j*, a measure of its impurity can be defined using the Gini index *i*(*t*):



At each step, the algorithm computes the impurity reduction provided by each site *s *with Δ*I*_*s *_= *i*(*t*) - *i*(*t*_1*s*_) - *i*(*t*_2*s*_), where *t*_1s _and *t*_2s _are the sub-nodes created when splitting with respect to *s*. The site *s *providing the largest impurity reduction is selected and the sequences at node *t *are split according to the different allelic states at this site. The splitting process stops when no significant gain in purity is obtained in adding more nodes.

According to their allelic state at the selected sites, each query sequence is first assigned to a leaf of the tree. The sequence is then assigned to the majority species in this leaf [20]. We ran this method using the R package *rpart *version 3 [25]. In this package, missing data are handled as follows: when the nucleotide at a splitting site is missing in a query sequence, the method finds the alternative site (with no missing data) that correlates the most with this site, and uses it instead as a splitting site.

### Random forest (RF)

This algorithm [26] overcomes some potential limitations of CART. Indeed, the first splitting site in CART influences subsequent splits, which may result in constraining the classification tree. To overcome this, the RF method generates a large number of trees from the learning sample by drawing subsets of *m *polymorphic site without replacement. Each subset is used to create a classification tree as in CART, which leads to a species assignment for a query sequence using the same majority rule as the CART method. Finally, the query sequence is assigned to the species indicated by the majority of trees.

We used the R package *randomForest *[27] to implement this method. We built 200 trees per data set. For real-life data sets, the number *m *of randomly selected polymorphic sites was optimized with the function *tuneRF *of *randomForest*. This procedure would have taken too long for the simulated data sets. Thus, for them, we used the same value of *m *for each set of simulations with given parameters. We tried various values of *m *ranging from  to *V/2*, where *V *is the number of polymorphic sites. We found that it was best to choose *V/2 *for small values of *V *and  for large values of *V*. Missing data were handled with the function *rfImpute *of *randomForest*. This method includes two steps: first, it replaces the missing nucleotide at a given site with the most frequent nucleotide at this site; second it performs *randomForest *on the resulting data and calculates a proximity matrix between individuals (based on the frequency at which pairs of individuals are in the same terminal node) and updates the value of the missing nucleotide of the individual with the nucleotide having the largest average proximity. This second step is iterated five times.

### Kernel methods

A kernel method [28] is an algorithm that projects data into a usually high-dimensional space and finds a hyperplane that aims to separate, as well as possible, data from each class (here the different species). The mapping of the original data to this new space is encoded by a kernel, here meaning a positive real-valued measure of pairwise similarity between data points. The effectiveness of kernel method algorithms is thus highly dependent on the choice of kernel as this determines separation of the classes in the new space. In the present context, kernel methods are relevant as we can define kernels to give pairwise comparisons between nucleotide sequences.

In this work, we proceeded by first retaining the set of polymorphic sites in the learning sample. We then used a contiguous subsequence kernel [29] defining similarity between sequences based on how many subsequences of length 1,2,..., *S *(where *S *is the number of polymorphic sites in the sequence) were not only shared between sequences but also aligned to the same set of positions in the global alignment. Intuitively, members of the same species should have more such common subsequences than members of different species. The contiguous subsequence kernel projects the learning set (nucleotide sequences) into a new (high-dimensional Euclidean) space and we then find a maximum-margin separating hyperplane using the SVM (Support Vector Machine) algorithm [28]. Query sequences are then projected into this space and classified based on which side of the hyperplane they fall on.

### Combining nuclear and cytoplasmic data

When information was available for one or several nuclear loci in addition to the cytoplasmic locus, the analysis was performed at each locus separately. Since nuclear loci are diploid, we assumed the two haplotypes of individuals at each nuclear locus to be known separately. The assignment of the query individual was performed through a voting procedure, in which the vote of each locus was weighted as follows. For NJ, PhyML and kernel methods, it was based on its proportion of correct assignment for the reference sample. For 1-NN, CART and RF, the weights were the probability for each locus of the query individual to belong to the various species.

## Results

### Simulated data sets

The supplementary table (Additional file 1) shows the success rate of the different methods (except kernel methods) for all parameter values when barcoding is performed with a single mitochondrial locus and no nuclear loci. We can see first that the performances of all methods were rather similar and some perform better than others depending on the situation. We see that among classification methods, 1-NN and RF generally performed better than CART, but not in all cases. Among phylogenetic methods, NJ performs slightly better than PhyML in most cases. When comparing the best phylogenetic method (NJ) and the best supervised classification methods (1-NN or RF, depending on the case), the difference was often slight, and sometimes not significant. 1-NN appeared to be the most reliable method, being always among the best performing ones.

This is seen more clearly in Tables 2 through 5 in which we present the success rates of the different methods when varying one parameter at a time, starting in all cases from the same model. This model involves a pair of species having diverged from each other *N*_f_/2 generations ago, where *N*_f _is the effective female population size. It is worth noting that an *N*_f_/2 separation time corresponds to half the expected coalescence time of two sequences in a standard Wright-Fisher model (selective neutrality, constant population size, panmixia). In this model, the size of the reference sample is 10. The results were examined for two values of *θ*: 3 and 30.

**Table 2:** Success rate (%) of data analysis methods with varying speciation time; mtDNA sequences were simulated for two species and a sample size *n *= 10; the mutation parameter *θ *was either 3 (A) or 30 (B).

**Speciation time**	**NJ**	**PhyML**	**1-NN**	**CART**	**RF**	**Kernel**	**P < 0.05**	**P < 0.01**
(A) *θ *= 3								
100	62.90	62.25 ¶	65.45 *	65.40	64.30	64.95	1	
500 †	87.25*	86.30	87.20	87.15	86.40	87.15		
1000	95.90	96.00	96.75 *	96.55	96.00	95.75		
5000	100.00 *	100.00 *	100.00 *	99.85	100.00 *	99.80		
10000	100.00 *	100.00 *	100.00 *	100.00 *	100.00 *	99.90		

(B) *θ *= 30								
100	75.60	75.30	76.25 *	75.50	77.75	73.45		
500 †	96.10	96.20 *	95.55	93.50 ¶	95.25	94.00 ¶	2	2
1000	99.15 *	99.15 *	98.55	97.10 ¶	98.35 ¶	96.90 ¶	3	3
5000	99.95	100.00 *	100.00 *	99.40 ¶	100.00 *	99.45 ¶	1	2
10000	100.00 *	100.00 *	100.00 *	99.40 ¶	100.00 *	99.55 ¶	2	2

* Best score

¶significantly below the best score. Columns 8 and 9 indicate the number of methods with *p*-values below 0.05 and 0.01 respectively.

† Focal set of parameters, for comparison across tables.

**Table 3:** Success rate (%) of data analysis methods when varying the reference sample size; mtDNA sequences were simulated for two species and a speciation time of T = 500 (*N*_*f*_/2); the mutation parameter *θ *was either 3 (A) or 30 (B).

**Reference** **sample size**	**NJ**	**PhyML**	**1-NN**	**CART**	**RF**	**Kernel**	**P < 0.05**	**P < 0.01**
(A) *θ *= 3								
3	77.45	77.50	78.05 *	77.15	77.35	76.15		
5	84.20 *	83.85	83.30	82.95	82.40	82.10		
10 †	87.25*	86.30	87.20	87.15	86.40	87.15		
25	92.00 *	91.70	91.10	90.80	89.40 ¶	90.75	1	1

(B) *θ *= 30								
3	82.80	82.95	83.55 *	79.40 ¶	81.95	80.45 ¶	2	2
5	89.50	89.45	90.25 *	86.20 ¶	89.30	86.85 ¶	2	2
10 †	96.10	96.20 *	95.55	93.50 ¶	95.25	94.00 ¶	1	1
25	98.95	98.95	99.15 *	98.30 ¶	99.00	98.20 ¶	2	1

* Best score

¶significantly below the best score. Columns 8 and 9 indicate the number of methods with *p*-values below 0.05 and 0.01 respectively.

† Focal set of parameters, for comparison across tables.

**Table 4:** Success rate (%) of data analysis methods for a number of species ranging from two to five; mtDNA sequences were simulated for a reference sample size *n *= 10 and a separation time *T *= 500 (*N*_*f*_/2); the mutation parameter *θ *was either 3 (A) or 30 (B).

**Number of species**	**NJ**	**PhyML**	**1-NN**	**CART**	**RF**	**Kernel**	**P < 0.05**	**P < 0.01**
(A) *θ *= 3								
2 †	87.25	86.30	87.30*	87.15	87.20	87.15		
3	81.73 *	80.77	80.67	80.40	80.97	81.10		
4	75.80	75.00	75.40	75.68	75.95 *	74.78 ¶	1	
5	73.26 *	72.36	72.58	72.84	73.22	70.74 ¶	1	1

(B) *θ *= 30								
2 †	96.10	96.20 *	95.55	93.50 ¶	95.25	94.00 ¶	2	2
3	94.40 *	94.23	94.00	90.93 ¶	93.50	92.10 ¶	2	2
4	93.78 *	93.73	92.90	90.10 ¶	92.53 ¶	91.40 ¶	3	2
5	92.46	92.38	92.70 *	88.98 ¶	92.08	90.46 ¶	2	2

* Best score

¶significantly below the best score. Columns 8 and 9 indicate the number of methods with *p*-values below 0.05 and 0.01 respectively.

† Focal set of parameters, for comparison across tables.

**Table 5:** Success rate (%) of data analysis methods for a number of additional nuclear loci; DNA sequences were simulated for two species, for a sample size *n *= 10 and a speciation time *T *= 500 (N/2); the mutation parameter *θ *was either 3 (A) or 30 (B).

**Number of** **nuclear loci (*n*_l_)**	**NJ**	**PhyML**	**1-NN**	**CART**	**RF**	**Kernel**	**P < 0.05**	**P < 0.01**
(A) *θ *= 3								
0 †	87.25	86.30	87.30*	87.15	86.40	87.15		
1	88.05 ¶	87.10 ¶	91.40 *	89.70	83.55 ¶	83.70 ¶	4	4
2	90.60 ¶	90.35 ¶	95.00 *	93.20 ¶	86.25 ¶	86.80 ¶	5	4
3	92.80 ¶	92.40 ¶	96.20 *	95.05	88.95 ¶	89.75 ¶	4	4
4	94.70 ¶	94.60 ¶	97.70 *	96.55 ¶	91.30 ¶	91.90 ¶	5	4

(B) *θ *= 30								
0 †	96.10	96.20 *	95.55	93.50 ¶	95.25	94.00 ¶	1	1
1	96.00	96.20	96.80	95.95	97.00*	96.10		
2	98.55*	98.55*	98.35	98.05	98.15	97.60		
3	99.40	99.30	99.50 *	98.95 ¶	99.40	98.75	1	1
4	99.75 *	99.70	99.75 *	99.50	99.75 *	99.50		

* Best score

¶significantly below the best score. Columns 8 and 9 indicate the number of methods with *p*-values below 0.05 and 0.01 respectively.

† Focal set of parameters, for comparison across tables.

The values obtained for this model are shown in each of Tables 2, 3, 4, 5 below (see e.g., Table 2, for rows "separation time = 500"). For this model, the performance of the various assignment methods ranged from an 86.30% to 87.25% success rate for *θ *= 3 and from a 93.50% to 96.20% success rate for *θ *= 30. The best method was NJ in the first case and PhyML in the second. It will be seen in the following that this result is not stable when changing simulation conditions.

### Varying speciation time

Results are shown in Table 2. For a speciation time of *N*_f_/10 (first line), all methods performed very poorly with a success rate under 66% for a small mutation rate (*θ *= 3). This separation time is very low and was designed as the lower boundary of the observations. Note however that all methods already reached a success rate of about 75% for this low speciation time when polymorphism was increased (*θ *= 30). Contrastingly, almost all methods showed a 100% success rate for very long separation times (*T *= 5*N*_f_, *T *= 10*N*_f_).

For intermediate conditions (T = *N*_f_/2 or *N*_f_), four methods had rather similar performances: NJ, PhyML, RF and 1-NN. CART and the kernel method also showed good performances for low *θ *values but were relatively less successful for high *θ *values. Overall, PhyML and NJ appeared to be the best methods for high *θ *values, and 1-NN the best method for low *θ *values.

### Varying sample size

Results are shown in Table 3. First, the success rate of all methods increased with sample size (*n*). This increase was sometimes quite large. For instance, for the 1-NN method with *θ *= 3, the success rate was only 78.35% for *n *= 3 but was 91.05% for *n *= 25. In general the best-performing method for the low *θ *value was NJ, but it was 1-NN for the high *θ *value. Overall, 1-NN appeared as the best method among those which did not significantly depart from the best score, as was already the case for varying speciation time.

### Increasing the number of speciation events

Few speciation events occur simultaneously. Simulating the simultaneous divergence of three to five species may thus seem somewhat unrealistic. However, taxonomic groups may undergo more or less extensive bursts of evolutionary radiations and we took this condition as a simple and replicable way to simulate this evolutionary context, which corresponds to a "worst-case scenario" for barcode analysis, as the lineages of all species may intermingle. Results are shown in Table 4. Increasing the number of species decreased the success rate of all methods. The decrease of the success rate was however much slower when *θ *was high than when it was low. The kernel method and CART appeared to be the methods most sensitive to an increased number of species: the decrease in their success rate with an increasing number of speciation events was the strongest. Again the other four methods had similar results, the NJ method performing slightly better than the others in a majority of cases.

### Adding nuclear loci

When a taxonomic case is difficult to resolve, a researcher may choose to sequence nuclear loci, which are genetically independent. We considered the effect of adding these nuclear loci. Results are shown in Table 5. The success of all assignment methods increased when the number of loci was increased. This increase was generally substantial, though not always. In particular, the RF method appeared quite poor at incorporating the information provided by the nuclear loci for the low *θ *value. For the low *θ *value, 1-NN was always the best method when at least one nuclear locus was included. All other methods were highly significantly less successful in almost all cases. For the high *θ *value, all methods performed about the same except for a single case of a significant difference for CART.

### Real-life data sets

These data sets combine all parameter variations as considered earlier, in a way which is difficult to systematically study with simulations. Thus, any of the barcoding drawbacks previously presented might occur, with the additional difficulties of the occurrence of missing data and of species with different population sizes. For the *Astraptes *data set (Table 6), all methods performed well with a success rate around 99%. For the two cowries data sets, the CART method was the least powerful. All other methods had a success rate above 90%, except PhyML and the kernel method for cowries at the subspecies level. The performance of all methods decreased for the cowries when subspecies were considered. The 1-NN and NJ methods performed the best for the species data set, while the RF method performed slightly better for the data set including subspecies. Similarly, almost all methods had similar success rates of above 90% for the three Amazonian butterfly data sets. CART and RF performed poorly for Ef1α. CART also performed poorly for the enlarged mtDNA sequence. The best performing method depended on the gene considered, although the 1-NN and NJ methods were always among the best, whilst the others did not perform well for at least one of the data sets.

**Table 6:** Success rate (%) of the methods on real data sets.

**Data set**	**NJ**	**PhyML**	**1-NN**	**CART**	**RF**	**Kernel**
*Astraptes*	99.36	99.36 *	99.36	98.22	99.36	99.57*
Cowries (species)	95.45 *	93.40	95.45 *	78.40	94.65	94.45
Cowries (species and subspecies)	91.10	86.37	91.31	72.38	91.41 *	89.20
Amazonian Butterflies (barcode)	91.73	91.20	90.40	75.47	92.00	92.80*
Amazonian Butterflies (mtDNA)	91.47	91.2	91.73	71.20	92.00	93.60*
Amazonian Butterflies (nuclear gene)	87.74	90.32 *	90.32 *	52.90	80.64	89.03

* Best score

## Discussion

### Comparison between methods

We aimed to compare the efficiency of six methods falling into two categories, which finally appear to constitute three groups: phylogenetic methods (NJ and PhyML), a simple distance-based method (1-NN) and three supervised statistical classification methods (CART, RF, kernel methods). In the simulation study, the success rates of the various methods were generally very close. The differences appeared significant in some cases because we ran a large number of simulations. However, these differences are often rather slight in magnitude. For half of the conditions surveyed in this study, no method significantly departed from the best performing one. Another striking result emerging from the simulation study is that best performing methods depended on the conditions. This had already been shown when comparing several distance and phylogenetic methods [16]. Here we show that it is still the case when comparing these methods with statistical classification methods.

More specifically, the simulation study shows that at low diversity (*θ *= 3), all methods perform approximately the same. On the other hand, when genetic diversity is high (*θ *= 30), distance methods (NJ, 1-NN) access best the information provided, except for large sample sizes (*n *= 25). This was the case over a wide range of speciation times and sample sizes and remained true when there was radiation between more than two species (Table 4). Under these conditions, CART and kernel methods constantly performed poorer than the others. RF was the only statistical classification method that had a similar success as the distance method (1-NN) or the phylogenetic methods (NJ, PhyML). The 1-NN method was the only one which never significantly departed from the best performing method, when it was not itself the best one. It thus appears to be the most reliable.

Among phylogenetic methods, NJ outperforms the maximum-likelihood method (PhyML) in most cases, as already observed by Elias et al. [5]. While algorithms like PhyML might be more efficient at resolving deep roots in phylogenetic trees, they seem to be less able to discriminate among recently separated taxa. The majority rule that we have applied here for the phylogenetic methods is quite rapid and allows us to perform comparisons on large quantities of simulated data. Note however that this method does not provide a confidence level for assignments. Some methods are available for providing such a confidence level [30-32]. These are all relatively time-intensive; we did not study them in our extensive simulation scheme, but users should consider them as they could provide an indication of the risk of false assignment of their sample.

Regarding the supervised statistical classification methods, while CART has some known limitations, we could have expected that more elaborate methods like RF or kernel methods would perform better than the simple 1-NN method. This result deserves examination. 1-NN merely states that the query belongs to the same species as the closest sequence, using some specified genetic distance. It should be noted that this principle applies well to the most difficult cases, those of Figures 1c and 1d, where a mutation is found in only one species but has not reached fixation. This case is related to the process of allele fixation in young species: at this step, some mutations are already "specific", but are not yet "diagnostic". Classification methods like CART and RF may be misled by mutations shared between species. Phylogenetic methods can also be misled because they will consider some closely related individuals belonging to other species. By focusing on the closest neighbour, 1-NN is less likely to be misled by individuals of a wrong species that would share many mutations with the query sequence. In this way, it is a kind of "cheap coalescent method". This may also explain why the 1-NN method, which considers only the closest neighbour, always performs better than methods that also consider more distant neighbours (2-NN, 3-NN, etc., unpublished results).

The supervised classification methods assume independence between the sequences being classified. Their performance strongly relies on how well the learning sample represents variability. These methods might be misled by small sample sizes (especially for high values of *θ*). In particular, kernel methods were relatively ineffective in our simulations. It could be that they do not deal easily with a number of simultaneous conditions. In particular, supervised learning classification methods such as kernel methods use the learning sample to determine a classification rule. These algorithms assume that the training set is representative of the whole dataset, but this is not always the case when the input space contains few observations from some species if the dimension is high (a few hundred sites, for instance). This may partially explain the lesser performance of kernel methods on simulated data. Furthermore, the performance of kernel methods is strongly conditioned by the choice of the kernel, and the purpose of this study was not to improve each method individually. Here, we used a standard kernel [29], so this method has a large potential for improvement, given a potentially better choice of kernel. A future development in this context could also be to combine different methods through an appropriate weighting procedure or aggregation method [33-35]. The kernel methods however performed well on most real data sets (giving the best result in 3 out of 6). As for the simulated data, the real data sets where the kernel method performed worse seem to be the cases where many classes are represented by few members, as for the cowries subspecies dataset.

Results on experimental data sets confirmed also that there is no best method on all data sets, even though the 1-NN method still seemed the most reliable. Real data sets are complex, due to an unknown history that may include gene flow between closely related species and demographic events like population expansions. This may explain why the methods performed differently. Choosing the best method for barcoding is thus a difficult problem. Estimating the *θ *value of the different species, for instance with [37] estimate, may provide some indication on the method that is likely to be the best for the data set in question. Nevertheless, since no method outperformed the others in all cases, a strategy when dealing with complex data sets may be to assess the performance of different methods on the reference sample set, using a leave-one-out procedure. The method performing the best may then be applied to the query dataset.

### Amount of data required

Besides the need to choose an appropriate method depending on the circumstances, our results clearly show that the performance of all methods clearly depends on the amount of available data. Barcode users can increase either i) the sample size, ii) the amount of information in the sequences by increasing their length or iii) the number of independent loci. For instance, for highly polymorphic species (*θ *= 30), with a sample size of 10, the success rate of most methods was above 95% provided that the species were separated for at least 0.5*N*_f _generations, where *N*_f _denotes the effective number of females in the species. For less polymorphic species (*θ *= 3), the same level of success was reached only when the separation time between species was above or equal to *N*_*f *_generations. This need for a high level of polymorphism increases strongly with the number of species considered (Table 4). If the COI sequences show too little polymorphism, extending their length might be useful.

Increasing the reference sample size had a strong positive effect on the success of methods: it increased from as much as 78% to 91% in certain cases (Table 2). Moreover, we considered species with no geographical structuring. For species with genetic structuring across their geographic distribution, increasing again the reference sample size with sampling in several locations would probably be of great help. Finally, for species for which increasing the sample size is not feasible, adding nuclear loci is an alternative possibility (Table 5). This has however a cost, since all individuals of the reference sample need to be genotyped for these additional loci. Nuclear loci also allow us to perform barcoding in cases where the COI locus is useless due to biological peculiarities, such as horizontal transfer and hitchhiking of mitochondria by Wolbachias [see e.g., [7]].

These patterns are confirmed by the study on real data sets. The good performance of all methods with the *Astraptes *data set can be explained by high separation and large sample sizes of these species, as is apparent on the phylogenetic tree from Hebert et al. [4], while in the cowries data set [6], the low level of separation and the lower sample sizes per species make the methods less efficient. The success rate is even lower for the cowries when considering the subspecies level since the separation times and the sample sizes are even smaller. For the Amazonian butterflies, we could not combine nuclear and cytoplasmic data sets as many individuals were not typed for both genes. Nevertheless, as noted by Elias et al. [5], we were also able to confirm that a nuclear gene EF1α can be used as additional information to the barcode.

## Conclusion

No one method used in this study appears to be the unique and universally best one for barcode data analysis. This is probably due to a variety of historical factors, such as history of speciation events, coalescent properties of the sample and stochasticity of mutation events, resulting in unpredictable data. This is reminiscent of what occurs in phylogenetics, for which no single method has been established as the best one for all cases, ever since Zuckerkandl and Pauling [36] discovered evolutionary clock scores. This suggests that choosing the best method for barcode interpretation must involve at least two steps. The first consists of an assessment of the methods conditional on the structure of the data. For instance, the efficiency of different methods can be evaluated on the reference sample set, which is generally well-characterized, and comprehensively sampled. In the context of this study, the 1-NN method appeared to be a very reliable method. The second step is to choose a method to apply to the query sequences. An alternative may involve further sequencing, either to sequence more mtDNA or to sequence loci from nuclear DNA. However, this choice is costly and contradicts the wishful universality of the standard barcode.

Finally, we note that for methodological purposes, we assumed that queries belonged to one of the species of the reference sample and used methods relevant to this assumption. However, only a fraction of all existing species have been identified by taxonomists. In more sophisticated applications of barcoding, the range of eligible species should not be bounded by the reference sample and DNA barcoding should aim to partition a collection of unresolved query sequences belonging to an unknown number of species.

## Competing interest 

The authors declare that they have no competing interests.

## Authors' contributions

The three leading investigators of this project were F. Austerlitz as a geneticist and a modeller, C. Laredo as a statistician and M. Veuille as a geneticist. These three undertook most of the writing of the article. F. Austerlitz designed the simulation procedure and obtained the experimental data from the various databases. He also performed the analyses on real and simulated data sets with the phylogenetic methods. O. David performed the analyses with CART, 1-NN and RF on the simulated data sets and designed the method to use both cytoplasmic and nuclear loci together. B. Schaeffer performed the basic data analyses and the analyses with CART, 1-NN and RF on the real data sets. K. Bleakley performed the analyses with kernel methods on the real and simulated data sets. R. Leblois and M. Olteanu contributed to discussions during advancement of the work.

## Supplementary Material

Additional file 1 Supplementary Table. Legend: Success rate of the different methods (except kernel methods) for all parameter sets tested Click here for file
