# Supplementary material for: DNA barcode analysis: a comparison of phylogenetic and statistical classification methods
Source: BMC Bioinformatics. 2009 Nov 10;10(Suppl 14):S10. doi: 10.1186/1471-2105-10-S14-S10 (PMC2775147; doi:10.1186/1471-2105-10-S14-S10)
Supplement: Additional file 1 — Supplementary Table. Legend: Success rate of the different methods (except kernel methods) for all parameter sets tested [file 1471-2105-10-S14-S10-S1.doc]

**Supplementary table: Success rate of the different methods (except kernel methods) for all parameter sets tested**

| mutation parameter(θ) | Number of species (n_S_) | Separation time (*T*) | sample size (n) | PhyML | NJ | CART | RF | NN |
| --- | --- | --- | --- | --- | --- | --- | --- | --- |
| 3 | 2 | 100 | 3 | 57.65% | 55.75% | 59.50% | 59.20% | 59.10% |
| 3 | 2 | 100 | 5 | 61.05% | 60.80% | 62.20% | 62.45% | 62.30% |
| 3 | 2 | 100 | 10 | 62.25% | 62.90% | 65.40% | 65.45% | 65.40% |
| 3 | 2 | 100 | 25 | 69.40% | 69.10% | 69.95% | 69.90% | 70.35% |
| 3 | 2 | 500 | 3 | 77.50% | 77.45% | 77.15% | 78.05% | 78.35% |
| 3 | 2 | 500 | 5 | 83.85% | 84.20% | 82.95% | 83.30% | 83.40% |
| 3 | 2 | 500 | 10 | 86.30% | 87.25% | 87.15% | 87.20% | 87.30% |
| 3 | 2 | 500 | 25 | 91.70% | 92.00% | 90.80% | 91.10% | 91.05% |
| 3 | 2 | 1000 | 3 | 89.15% | 89.05% | 87.20% | 89.10% | 89% |
| 3 | 2 | 1000 | 5 | 92.65% | 93.30% | 92.10% | 92.65% | 92.75% |
| 3 | 2 | 1000 | 10 | 96.00% | 95.90% | 96.55% | 96.75% | 96.70% |
| 3 | 2 | 1000 | 25 | 97.30% | 97.45% | 97.50% | 97.80% | 97.85% |
| 3 | 2 | 5000 | 3 | 99.70% | 99.60% | 98.15% | 99.65% | 99.70% |
| 3 | 2 | 5000 | 5 | 99.90% | 99.90% | 99.20% | 99.90% | 99.95% |
| 3 | 2 | 5000 | 10 | 100.00% | 100.00% | 99.85% | 100.00% | 100% |
| 3 | 2 | 5000 | 25 | 100.00% | 100.00% | 99.95% | 100.00% | 100% |
| 3 | 2 | 10000 | 3 | 100.00% | 99.60% | 99.20% | 100.00% | 100% |
| 3 | 2 | 10000 | 5 | 99.95% | 100.00% | 99.60% | 100.00% | 100% |
| 3 | 2 | 10000 | 10 | 100.00% | 100.00% | 99.75% | 100.00% | 100% |
| 3 | 2 | 10000 | 25 | 100.00% | 100.00% | 100% | 100.00% | 100% |
| 3 | 3 | 100 | 3 | 42.00% | 42.27% | 42.30% | 42.73% | 42.50% |
| 3 | 3 | 100 | 5 | 45.17% | 45.57% | 44.40% | 44.53% | 43.93% |
| 3 | 3 | 100 | 10 | 50.30% | 50.47% | 50.67% | 50.77% | 50.83% |
| 3 | 3 | 100 | 25 | 53.93% | 54.17% | 53.40% | 53.57% | 53.40% |
| 3 | 3 | 500 | 3 | 67.53% | 68.60% | 67.87% | 68.27% | 68.10% |
| 3 | 3 | 500 | 5 | 74.63% | 75.80% | 74.17% | 74.80% | 74.23% |
| 3 | 3 | 500 | 10 | 80.77% | 81.73% | 80.40% | 80.97% | 80.67% |
| 3 | 3 | 500 | 25 | 86.57% | 86.83% | 86.70% | 86.53% | 86.47% |
| 3 | 3 | 1000 | 3 | 85.23% | 86.23% | 82.93% | 83.80% | 83.80% |
| 3 | 3 | 1000 | 5 | 89.57% | 90.40% | 88.07% | 88.67% | 88.13% |
| 3 | 3 | 1000 | 10 | 94.27% | 94.70% | 93.37% | 93.87% | 93.27% |
| 3 | 3 | 1000 | 25 | 96.43% | 96.57% | 95.93% | 96.13% | 95.80% |
| 3 | 3 | 5000 | 3 | 99.50% | 99.57% | 97.57% | 99.67% | 99.73% |
| 3 | 3 | 5000 | 5 | 99.90% | 99.93% | 98.93% | 99.70% | 99.87% |
| 3 | 3 | 5000 | 10 | 99.97% | 99.97% | 99.87% | 99.93% | 99.97% |
| 3 | 3 | 5000 | 25 | 100.00% | 100.00% | 99.93% | 100.00% | 100% |
| 3 | 3 | 10000 | 3 | 100.00% | 100.00% | 98.37% | 100.00% | 100% |
| 3 | 3 | 10000 | 5 | 100.00% | 100.00% | 99.20% | 100.00% | 100% |
| 3 | 3 | 10000 | 10 | 99.97% | 99.97% | 99.73% | 100.00% | 100% |
| 3 | 3 | 10000 | 25 | 100.00% | 100.00% | 100% | 100.00% | 100% |
| 3 | 4 | 100 | 3 | 34.50% | 35.23% | 34.52% | 34.60% | 34.80% |
| 3 | 4 | 100 | 5 | 36.80% | 37.13% | 36.80% | 37.18% | 36.88% |
| 3 | 4 | 100 | 10 | 41.33% | 41.88% | 42.83% | 42.75% | 42.33% |
| 3 | 4 | 100 | 25 | 46.38% | 46.35% | 46.30% | 45.88% | 46.08% |
| 3 | 4 | 500 | 3 | 61.20% | 62.28% | 61.95% | 62.35% | 62% |
| 3 | 4 | 500 | 5 | 69.53% | 70.75% | 69.85% | 70.28% | 69.60% |
| 3 | 4 | 500 | 10 | 75.00% | 75.80% | 75.67% | 75.95% | 75.40% |
| 3 | 4 | 500 | 25 | 82.83% | 83.23% | 81.47% | 81.53% | 81.62% |
| 3 | 4 | 1000 | 3 | 80.20% | 81.58% | 79.22% | 80.33% | 80.27% |
| 3 | 4 | 1000 | 5 | 87.25% | 88.03% | 85.47% | 86.58% | 85.80% |
| 3 | 4 | 1000 | 10 | 91.83% | 92.63% | 91.65% | 92.12% | 91.60% |
| 3 | 4 | 1000 | 25 | 95.48% | 95.65% | 94.40% | 94.45% | 94.33% |
| 3 | 4 | 5000 | 3 | 99.45% | 99.45% | 97.10% | 99.52% | 99.48% |
| 3 | 4 | 5000 | 5 | 99.80% | 99.83% | 98.95% | 99.83% | 99.75% |
| 3 | 4 | 5000 | 10 | 99.83% | 99.85% | 99.58% | 99.98% | 99.98% |
| 3 | 4 | 5000 | 25 | 99.98% | 99.98% | 99.92% | 99.98% | 99.98% |
| 3 | 4 | 10000 | 3 | 99.95% | 99.98% | 98.42% | 100.00% | 100% |
| 3 | 4 | 10000 | 5 | 100.00% | 100.00% | 99.35% | 100.00% | 100% |
| 3 | 4 | 10000 | 10 | 100.00% | 100.00% | 99.58% | 100.00% | 100% |
| 3 | 4 | 10000 | 25 | 100.00% | 100.00% | 99.98% | 100.00% | 100% |
| 3 | 5 | 100 | 3 | 28.68% | 29.30% | 28.92% | 28.70% | 28.96% |
| 3 | 5 | 100 | 5 | 31.86% | 32.10% | 31.78% | 31.86% | 31.54% |
| 3 | 5 | 100 | 10 | 34.78% | 35.30% | 37.14% | 37.12% | 37.42% |
| 3 | 5 | 100 | 25 | 40.64% | 41.02% | 42.60% | 42.44% | 42.96% |
| 3 | 5 | 500 | 3 | 57.38% | 59.02% | 57.78% | 57.92% | 57.06% |
| 3 | 5 | 500 | 5 | 63.90% | 65.42% | 64.68% | 64.92% | 64.12% |
| 3 | 5 | 500 | 10 | 72.36% | 73.24% | 72.84% | 73.22% | 72.58% |
| 3 | 5 | 500 | 25 | 78.58% | 78.66% | 79.06% | 78.98% | 78.88% |
| 3 | 5 | 1000 | 3 | 77.32% | 78.94% | 77.48% | 78.92% | 78.10% |
| 3 | 5 | 1000 | 5 | 84.06% | 85.14% | 82.72% | 83.98% | 83.32% |
| 3 | 5 | 1000 | 10 | 90.28% | 91.16% | 90.46% | 90.84% | 90.32% |
| 3 | 5 | 1000 | 25 | 93.56% | 93.74% | 93.74% | 93.74% | 93.58% |
| 3 | 5 | 5000 | 3 | 99.52% | 99.60% | 97.20% | 99.56% | 99.42% |
| 3 | 5 | 5000 | 5 | 99.56% | 99.62% | 98.72% | 99.72% | 99.88% |
| 3 | 5 | 5000 | 10 | 99.94% | 99.94% | 99.56% | 99.84% | 99.84% |
| 3 | 5 | 5000 | 25 | 99.96% | 99.96% | 99.84% | 99.96% | 99.98% |
| 3 | 5 | 10000 | 3 | 100.00% | 100.00% | 98.64% | 100.00% | 99.98% |
| 3 | 5 | 10000 | 5 | 100.00% | 100.00% | 99.22% | 100.00% | 100% |
| 3 | 5 | 10000 | 10 | 100.00% | 100.00% | 99.72% | 100.00% | 100% |
| 3 | 5 | 10000 | 25 | 100.00% | 100.00% | 99.96% | 100.00% | 100% |
| 12 | 2 | 100 | 3 | 60.40% | 61.00% | 61.70% | 62.00% | 61.40% |
| 12 | 2 | 100 | 5 | 66.15% | 66.35% | 65.75% | 66.40% | 66.05% |
| 12 | 2 | 100 | 10 | 71.30% | 71.90% | 72.55% | 73.00% | 72.80% |
| 12 | 2 | 100 | 25 | 79.80% | 80.15% | 80.35% | 80.60% | 80.95% |
| 12 | 2 | 500 | 3 | 83.15% | 83.55% | 80.40% | 82.70% | 82.50% |
| 12 | 2 | 500 | 5 | 89.15% | 89.80% | 87.00% | 87.90% | 88.45% |
| 12 | 2 | 500 | 10 | 94.85% | 94.45% | 93.30% | 94.60% | 94.40% |
| 12 | 2 | 500 | 25 | 99.15% | 99.15% | 97.90% | 98.40% | 98.15% |
| 12 | 2 | 1000 | 3 | 92.40% | 92.60% | 88.85% | 90.70% | 90.85% |
| 12 | 2 | 1000 | 5 | 95.55% | 95.70% | 93.80% | 95.60% | 96.20% |
| 12 | 2 | 1000 | 10 | 98.60% | 98.50% | 97.20% | 98.50% | 98.60% |
| 12 | 2 | 1000 | 25 | 99.65% | 99.65% | 99.30% | 99.70% | 99.80% |
| 12 | 2 | 5000 | 3 | 99.95% | 100.00% | 98.10% | 99.80% | 99.80% |
| 12 | 2 | 5000 | 5 | 99.90% | 99.90% | 99.05% | 100.00% | 100.00% |
| 12 | 2 | 5000 | 10 | 100.00% | 100.00% | 99.50% | 100.00% | 100.00% |
| 12 | 2 | 5000 | 25 | 100.00% | 100.00% | 99.90% | 100.00% | 100.00% |
| 12 | 2 | 10000 | 3 | 100.00% | 100.00% | 98.25% | 100.00% | 100.00% |
| 12 | 2 | 10000 | 5 | 100.00% | 100.00% | 99.05% | 100.00% | 100.00% |
| 12 | 2 | 10000 | 10 | 100.00% | 100.00% | 99.80% | 100.00% | 100.00% |
| 12 | 2 | 10000 | 25 | 100.00% | 100.00% | 100.00% | 100.00% | 100.00% |
| 12 | 3 | 100 | 3 | 45.20% | 45.30% | 46.77% | 47.33% | 47.67% |
| 12 | 3 | 100 | 5 | 52.63% | 53.23% | 52.53% | 52.77% | 53.43% |
| 12 | 3 | 100 | 10 | 61.63% | 62.43% | 61.70% | 61.83% | 61.60% |
| 12 | 3 | 100 | 25 | 72.27% | 73.40% | 73.07% | 73.40% | 72.70% |
| 12 | 3 | 500 | 3 | 76.63% | 76.83% | 72.77% | 76.20% | 76.80% |
| 12 | 3 | 500 | 5 | 84.73% | 85.00% | 82.30% | 84.23% | 84.57% |
| 12 | 3 | 500 | 10 | 92.63% | 92.93% | 90.60% | 92.03% | 92.10% |
| 12 | 3 | 500 | 25 | 98.30% | 98.40% | 97.33% | 97.80% | 97.97% |
| 12 | 3 | 1000 | 3 | 88.37% | 88.50% | 85.63% | 88.20% | 89.00% |
| 12 | 3 | 1000 | 5 | 93.17% | 93.07% | 92.00% | 93.77% | 94.23% |
| 12 | 3 | 1000 | 10 | 97.77% | 97.87% | 96.80% | 98.20% | 98.27% |
| 12 | 3 | 1000 | 25 | 99.47% | 99.37% | 99.30% | 99.60% | 99.53% |
| 12 | 3 | 5000 | 3 | 99.83% | 99.83% | 96.93% | 99.70% | 99.73% |
| 12 | 3 | 5000 | 5 | 99.83% | 99.83% | 98.13% | 99.97% | 99.97% |
| 12 | 3 | 5000 | 10 | 99.97% | 99.97% | 99.33% | 99.93% | 99.97% |
| 12 | 3 | 5000 | 25 | 100.00% | 100.00% | 99.97% | 100.00% | 100.00% |
| 12 | 3 | 10000 | 3 | 100.00% | 100.00% | 98.03% | 100.00% | 100.00% |
| 12 | 3 | 10000 | 5 | 100.00% | 100.00% | 99.00% | 100.00% | 100.00% |
| 12 | 3 | 10000 | 10 | 100.00% | 100.00% | 99.67% | 100.00% | 100.00% |
| 12 | 3 | 10000 | 25 | 100.00% | 100.00% | 99.93% | 100.00% | 100.00% |
| 12 | 4 | 100 | 3 | 39.95% | 40.30% | 39.10% | 39.55% | 38.85% |
| 12 | 4 | 100 | 5 | 44.23% | 45.00% | 44.70% | 45.25% | 45.10% |
| 12 | 4 | 100 | 10 | 54.90% | 54.95% | 54.43% | 54.77% | 54.05% |
| 12 | 4 | 100 | 25 | 66.33% | 66.43% | 66.72% | 66.72% | 66.33% |
| 12 | 4 | 500 | 3 | 73.48% | 74.13% | 68.33% | 71.05% | 71.55% |
| 12 | 4 | 500 | 5 | 81.05% | 81.95% | 80.45% | 82.80% | 82.83% |
| 12 | 4 | 500 | 10 | 91.38% | 91.93% | 89.58% | 91.27% | 91.15% |
| 12 | 4 | 500 | 25 | 97.35% | 97.53% | 96.35% | 97.12% | 97.10% |
| 12 | 4 | 1000 | 3 | 87.45% | 87.75% | 82.78% | 86.62% | 87.40% |
| 12 | 4 | 1000 | 5 | 93.60% | 93.58% | 89.92% | 92.85% | 93.08% |
| 12 | 4 | 1000 | 10 | 97.68% | 97.70% | 96.33% | 97.32% | 97.35% |
| 12 | 4 | 1000 | 25 | 99.35% | 99.33% | 99.00% | 99.38% | 99.45% |
| 12 | 4 | 5000 | 3 | 99.70% | 99.70% | 96.33% | 99.62% | 99.75% |
| 12 | 4 | 5000 | 5 | 99.95% | 99.95% | 98.67% | 99.80% | 99.85% |
| 12 | 4 | 5000 | 10 | 99.95% | 99.95% | 99.35% | 99.92% | 99.95% |
| 12 | 4 | 5000 | 25 | 100.00% | 100.00% | 99.88% | 100.00% | 100.00% |
| 12 | 4 | 10000 | 3 | 99.98% | 99.98% | 97.97% | 100.00% | 100.00% |
| 12 | 4 | 10000 | 5 | 99.98% | 100.00% | 98.83% | 100.00% | 100.00% |
| 12 | 4 | 10000 | 10 | 100.00% | 100.00% | 99.65% | 100.00% | 100.00% |
| 12 | 4 | 10000 | 25 | 100.00% | 100.00% | 99.80% | 100.00% | 100.00% |
| 12 | 5 | 100 | 3 | 34.52% | 35.10% | 34.60% | 34.80% | 34.84% |
| 12 | 5 | 100 | 5 | 39.94% | 39.86% | 39.54% | 39.88% | 39.30% |
| 12 | 5 | 100 | 10 | 49.66% | 50.70% | 50.60% | 51.08% | 50.52% |
| 12 | 5 | 100 | 25 | 61.36% | 62.44% | 62.50% | 62.56% | 62.14% |
| 12 | 5 | 500 | 3 | 70.16% | 70.98% | 66.40% | 69.76% | 71.18% |
| 12 | 5 | 500 | 5 | 79.08% | 80.06% | 77.00% | 80.16% | 80.22% |
| 12 | 5 | 500 | 10 | 90.48% | 90.80% | 88.18% | 89.90% | 90.24% |
| 12 | 5 | 500 | 25 | 97.08% | 97.34% | 96.32% | 97.06% | 96.90% |
| 12 | 5 | 1000 | 3 | 86.82% | 87.40% | 81.40% | 85.74% | 86.48% |
| 12 | 5 | 1000 | 5 | 92.70% | 93.04% | 89.62% | 92.88% | 92.64% |
| 12 | 5 | 1000 | 10 | 97.14% | 97.30% | 95.70% | 97.14% | 97.06% |
| 12 | 5 | 1000 | 25 | 99.34% | 99.34% | 98.86% | 99.44% | 99.42% |
| 12 | 5 | 5000 | 3 | 99.60% | 99.60% | 96.12% | 99.76% | 99.80% |
| 12 | 5 | 5000 | 5 | 99.92% | 99.94% | 98.24% | 99.80% | 99.82% |
| 12 | 5 | 5000 | 10 | 99.94% | 99.92% | 99.22% | 99.94% | 99.96% |
| 12 | 5 | 5000 | 25 | 100.00% | 100.00% | 99.68% | 100.00% | 100.00% |
| 12 | 5 | 10000 | 3 | 99.98% | 99.98% | 97.82% | 99.98% | 99.98% |
| 12 | 5 | 10000 | 5 | 100.00% | 100.00% | 98.74% | 100.00% | 100.00% |
| 12 | 5 | 10000 | 10 | 100.00% | 100.00% | 99.50% | 100.00% | 100.00% |
| 12 | 5 | 10000 | 25 | 100.00% | 100.00% | 99.80% | 100.00% | 100.00% |
| 30 | 2 | 100 | 3 | 60.50% | 60.50% | 61.50% | 61.60% | 61.10% |
| 30 | 2 | 100 | 5 | 67.80% | 67.30% | 65.25% | 65.85% | 65.45% |
| 30 | 2 | 100 | 10 | 75.30% | 75.60% | 75.50% | 77.75% | 76.25% |
| 30 | 2 | 100 | 25 | 86.20% | 87.20% | 85.15% | 86.10% | 86.40% |
| 30 | 2 | 500 | 3 | 82.95% | 82.80% | 79.40% | 81.95% | 83.55% |
| 30 | 2 | 500 | 5 | 89.45% | 89.50% | 86.20% | 89.30% | 90.25% |
| 30 | 2 | 500 | 10 | 96.20% | 96.10% | 93.50% | 95.25% | 95.55% |
| 30 | 2 | 500 | 25 | 98.95% | 98.95% | 98.30% | 99.00% | 99.15% |
| 30 | 2 | 1000 | 3 | 93.75% | 93.65% | 88.15% | 91.95% | 92.70% |
| 30 | 2 | 1000 | 5 | 96.55% | 96.60% | 93.45% | 95.05% | 96.05% |
| 30 | 2 | 1000 | 10 | 99.15% | 99.15% | 97.10% | 98.35% | 98.55% |
| 30 | 2 | 1000 | 25 | 99.70% | 99.70% | 99.35% | 99.50% | 99.55% |
| 30 | 2 | 5000 | 3 | 100.00% | 100.00% | 97.25% | 99.85% | 99.85% |
| 30 | 2 | 5000 | 5 | 99.90% | 99.90% | 98.50% | 100.00% | 100.00% |
| 30 | 2 | 5000 | 10 | 100.00% | 99.95% | 99.40% | 100.00% | 100.00% |
| 30 | 2 | 5000 | 25 | 100.00% | 100.00% | 99.80% | 100.00% | 100.00% |
| 30 | 2 | 10000 | 3 | 100.00% | 100.00% | 97.90% | 100.00% | 100.00% |
| 30 | 2 | 10000 | 5 | 100.00% | 100.00% | 98.85% | 100.00% | 100.00% |
| 30 | 2 | 10000 | 10 | 100.00% | 100.00% | 99.40% | 100.00% | 100.00% |
| 30 | 2 | 10000 | 25 | 100.00% | 100.00% | 99.70% | 100.00% | 100.00% |
| 30 | 3 | 100 | 3 | 48.13% | 48.80% | 48.20% | 49.40% | 49.40% |
| 30 | 3 | 100 | 5 | 55.93% | 56.10% | 52.83% | 53.47% | 53.90% |
| 30 | 3 | 100 | 10 | 65.93% | 66.37% | 66.80% | 68.63% | 68.47% |
| 30 | 3 | 100 | 25 | 81.60% | 82.13% | 79.10% | 80.27% | 80.57% |
| 30 | 3 | 500 | 3 | 77.53% | 77.50% | 73.30% | 76.67% | 77.63% |
| 30 | 3 | 500 | 5 | 86.07% | 86.20% | 82.40% | 85.70% | 86.67% |
| 30 | 3 | 500 | 10 | 94.23% | 94.40% | 90.93% | 93.50% | 94.17% |
| 30 | 3 | 500 | 25 | 98.53% | 98.60% | 97.57% | 98.70% | 98.63% |
| 30 | 3 | 1000 | 3 | 89.80% | 89.93% | 86.03% | 90.07% | 90.97% |
| 30 | 3 | 1000 | 5 | 94.93% | 95.03% | 91.77% | 94.47% | 95.07% |
| 30 | 3 | 1000 | 10 | 98.03% | 98.10% | 97.00% | 98.37% | 98.40% |
| 30 | 3 | 1000 | 25 | 99.80% | 99.80% | 99.17% | 99.60% | 99.60% |
| 30 | 3 | 5000 | 3 | 99.67% | 99.70% | 96.53% | 99.73% | 99.87% |
| 30 | 3 | 5000 | 5 | 99.83% | 99.83% | 98.27% | 99.93% | 99.97% |
| 30 | 3 | 5000 | 10 | 99.93% | 99.93% | 98.90% | 99.97% | 99.93% |
| 30 | 3 | 5000 | 25 | 100.00% | 100.00% | 99.43% | 99.97% | 100.00% |
| 30 | 3 | 10000 | 3 | 100.00% | 100.00% | 96.80% | 100.00% | 100.00% |
| 30 | 3 | 10000 | 5 | 100.00% | 100.00% | 98.17% | 100.00% | 100.00% |
| 30 | 3 | 10000 | 10 | 100.00% | 100.00% | 99.03% | 100.00% | 100.00% |
| 30 | 3 | 10000 | 25 | 100.00% | 100.00% | 99.70% | 100.00% | 100.00% |
| 30 | 4 | 100 | 3 | 41.55% | 41.78% | 39.52% | 41.05% | 40.65% |
| 30 | 4 | 100 | 5 | 47.23% | 47.98% | 47.35% | 49.65% | 49.68% |
| 30 | 4 | 100 | 10 | 61.58% | 62.03% | 58.98% | 60.30% | 61.18% |
| 30 | 4 | 100 | 25 | 76.30% | 77.28% | 75.70% | 76.98% | 76.65% |
| 30 | 4 | 500 | 3 | 74.78% | 74.65% | 69.70% | 73.98% | 75.00% |
| 30 | 4 | 500 | 5 | 84.18% | 84.58% | 79.75% | 84.35% | 85.15% |
| 30 | 4 | 500 | 10 | 93.73% | 93.78% | 90.10% | 92.53% | 92.90% |
| 30 | 4 | 500 | 25 | 98.45% | 98.40% | 97.52% | 98.47% | 98.50% |
| 30 | 4 | 1000 | 3 | 89.25% | 89.30% | 81.80% | 87.10% | 88.22% |
| 30 | 4 | 1000 | 5 | 93.33% | 93.25% | 90.38% | 93.40% | 94.00% |
| 30 | 4 | 1000 | 10 | 97.98% | 97.95% | 95.33% | 97.38% | 97.55% |
| 30 | 4 | 1000 | 25 | 99.54% | 99.63% | 98.78% | 99.55% | 99.58% |
| 30 | 4 | 5000 | 3 | 99.75% | 99.75% | 95.45% | 99.75% | 99.92% |
| 30 | 4 | 5000 | 5 | 99.90% | 99.90% | 97.30% | 99.92% | 99.90% |
| 30 | 4 | 5000 | 10 | 99.98% | 99.98% | 98.67% | 99.95% | 99.98% |
| 30 | 4 | 5000 | 25 | 100.00% | 100.00% | 99.48% | 100.00% | 100.00% |
| 30 | 4 | 10000 | 3 | 100.00% | 100.00% | 96.78% | 100.00% | 99.98% |
| 30 | 4 | 10000 | 5 | 100.00% | 100.00% | 98.05% | 100.00% | 100.00% |
| 30 | 4 | 10000 | 10 | 100.00% | 100.00% | 99.15% | 100.00% | 100.00% |
| 30 | 4 | 10000 | 25 | 100.00% | 100.00% | 99.58% | 100.00% | 100.00% |
| 30 | 5 | 100 | 3 | 38.22% | 38.28% | 35.46% | 36.30% | 36.72% |
| 30 | 5 | 100 | 5 | 46.34% | 47.10% | 43.48% | 44.90% | 44.92% |
| 30 | 5 | 100 | 10 | 56.00% | 56.74% | 55.48% | 57.04% | 56.90% |
| 30 | 5 | 100 | 25 | 74.64% | 75.66% | 74.34% | 75.72% | 75.08% |
| 30 | 5 | 500 | 3 | 73.00% | 73.02% | 66.34% | 72.12% | 73.14% |
| 30 | 5 | 500 | 5 | 82.30% | 82.52% | 77.88% | 82.76% | 83.36% |
| 30 | 5 | 500 | 10 | 92.38% | 92.46% | 88.98% | 92.08% | 92.70% |
| 30 | 5 | 500 | 25 | 97.72% | 97.72% | 96.90% | 97.90% | 98.18% |
| 30 | 5 | 1000 | 3 | 88.34% | 88.20% | 81.54% | 87.26% | 88.42% |
| 30 | 5 | 1000 | 5 | 93.24% | 93.14% | 88.72% | 93.20% | 93.54% |
| 30 | 5 | 1000 | 10 | 97.12% | 97.14% | 95.44% | 97.74% | 98.00% |
| 30 | 5 | 1000 | 25 | 99.42% | 99.42% | 98.84% | 99.52% | 99.52% |
| 30 | 5 | 5000 | 3 | 99.84% | 99.78% | 94.72% | 99.84% | 99.96% |
| 30 | 5 | 5000 | 5 | 99.88% | 99.86% | 97.24% | 99.84% | 99.86% |
| 30 | 5 | 5000 | 10 | 100.00% | 100.00% | 98.88% | 99.98% | 99.96% |
| 30 | 5 | 5000 | 25 | 99.98% | 99.98% | 99.62% | 100.00% | 100.00% |
| 30 | 5 | 10000 | 3 | 100.00% | 100.00% | 95.84% | 100.00% | 99.98% |
| 30 | 5 | 10000 | 5 | 100.00% | 100.00% | 97.48% | 100.00% | 100.00% |
| 30 | 5 | 10000 | 10 | 100.00% | 100.00% | 99.00% | 100.00% | 100.00% |
| 30 | 5 | 10000 | 25 | 100.00% | 100.00% | 99.50% | 100.00% | 100.00% |
